# Smart Materials Prediction: Applying Machine Learning to Lithium Solid-State Electrolyte

**DOI:** 10.3390/ma15031157

**Published:** 2022-02-02

**Authors:** Qianyu Hu, Kunfeng Chen, Fei Liu, Mengying Zhao, Feng Liang, Dongfeng Xue

**Affiliations:** 1 Institute of Novel Semiconductors, State Key Laboratory of Crystal Materials, Shandong University, Jinan 250100, China; 202112795@mail.sdu.edu.cn (Q.H.); zmy418@sdu.edu.cn (M.Z.); 2 Wuhan Institute of Marine Electric Propulsion, CSIC, Wuhan 430064, China; liuf.zkdlkrgb@gmail.com; 3 State Key Laboratory of Complex Non-Ferrous Metal Resources Clean Application, Faculty of Metallurgical and Energy Engineering, Kunming University of Science and Technology, Kunming 650093, China; liangfeng@kust.edu.cn; 4 Multiscale Crystal Materials Research Center, Shenzhen Institute of Advanced Technology, Chinese Academy of Sciences, Shenzhen 518055, China

**Keywords:** machine learning, solid state electrolyte, new materials discovery, lithium battery

## Abstract

Traditionally, the discovery of new materials has often depended on scholars’ computational and experimental experience. The traditional trial-and-error methods require many resources and computing time. Due to new materials’ properties becoming more complex, it is difficult to predict and identify new materials only by general knowledge and experience. Material prediction tools based on machine learning (ML) have been successfully applied to various materials fields; they are beneficial for modeling and accelerating the prediction process for materials that cannot be accurately predicted. However, the obstacles of disciplinary span led to many scholars in materials not having complete knowledge of data-driven materials science methods. This paper provides an overview of the general process of ML applied to materials prediction and uses solid-state electrolytes (SSE) as an example. Recent approaches and specific applications to ML in the materials field and the requirements for building ML models for predicting lithium SSE are reviewed. Finally, some current obstacles to applying ML in materials prediction and prospects are described with the expectation that more materials scholars will be aware of the application of ML in materials prediction.

## 1. Introduction

Materials science often focuses on the study of materials’ processing, properties and applications. Since ancient times, materials scientists have wanted to predict and apply materials from scratch [1]. The traditional way of deploying new materials is through the experience of materials scientists who gather and perform theoretical calculations and experimental confirmation, which is inefficient, resource-intensive and expensive in today’s information explosion. The fierce competition in the manufacturing industry and the rapid economic development of the sector have posed a new challenge to scholars in materials science: how to shorten the product and market application cycle of new materials.

Since the 1990s, the integration and intelligence of large-scale data using computers have become a topic of great interest. As an essential branch of artificial intelligence, ML has been applied with great success in various fields such as psychological science [2], earth science [3], biomedicine [4] and communication technology [5]. The combination of big data and artificial intelligence has been called the “fourth paradigm of science” [6]. To date, ML has also been widely used in predicting novel materials; ML at its core is a statistical algorithm, which is the same as the researcher’s thinking but much faster than the researcher’s intuitive predictions [7]. The ML can also significantly reduce the prediction time and accelerate the prediction process for the traditional input structure whose properties are calculated by approximating the Schrödinger equation to solve the linear computation, for example, Wang et al. predicted new materials with reduction of about 95 years by ML assisted analysis [8].

How does ML improve statistical speed to accelerate the computational cycle of predicting new materials? The main reason is that it is not like traditional computing methods that generally use hard-coded algorithms provided by human experts but based on a large amount of data and specific algorithmic rules so that the computer can simulate the human learning process and through learning to make intelligent decisions to achieve the purpose of the final prediction. The learning process of humans is firstly to accumulate knowledge, summarize the experience and obtain the laws, optimize and construct the model of their knowledge theory system and, finally, reach the degree of flexible application and even innovation. ML applied to materials science is almost the same as the logic of human thinking.

The first stage is knowledge accumulation, i.e., data collection. The adequacy of the data set often dramatically affects the construction and application of the algorithm model. The original collected data set often have different forms. We need to process the original data set to obtain its main features and convert it into a data format that is more suitable for the constructed model, which we call descriptors (fingerprints). The more compatible features are certainly more beneficial to our prediction process for new materials. This pre-processing process of obtaining data features is called feature engineering. After we have got enough material features, we must learn the fingerprints we have received. The so-called model learning process uses specific algorithms to analyze the data fingerprints, which are used to explore the implied relationships among data. We can train the model by increasing the data set and evaluating the model’s accuracy according to the training results, optimizing the algorithm model according to its evaluation and finally, using the optimized optimal model to analyze and predict the unknown materials.

**Figure 1:**
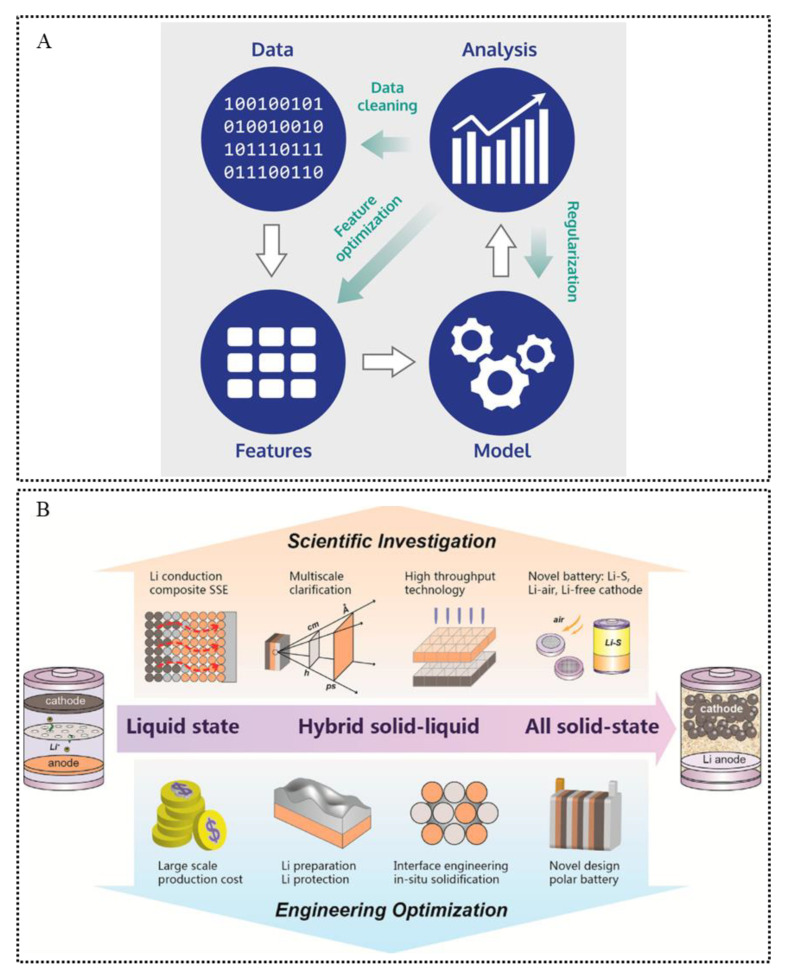
(**A**) Key steps in building a ML model. The white arrows indicate the data flow; green arrows indicate actions that can be identified and performed after analysis to improve the model’s performance. Reprinted from Reference [9] with permission from John Wiley and Sons. (**B**) Perspectives for future studies on solid-state batteries. Reprinted from Reference [13] with permission from American Chemical Society.

In shortly, the ML applied in the material field mainly consist of following steps: data acquisition, feature engineering, model construction, analysis and the targeted injection of new data for optimization progress [9] and, finally, form a complete and self-consistent system (Figure 1A), which can be continuously and adaptively improved and ultimately achieve the purpose of predicting new materials.

Lithium-ion batteries (LIBs), as representatives of modern high-performance batteries, are now widely used in our lives, ranging from aerospace to small applications in personal electronics [10]. The present LIBs use liquid organic electrolytes, which often results in safety hazards [11]. The development of more advanced energy storage technologies is one of the significant trends in energy storage field. Scholars are exploring SSE with high ionic conductivity, high mechanical strength and non-flammability [12] and expecting to be able to apply it to all-solid-state batteries [13] (Figure 1B).

The earliest discovery of fast lithium-ion conducting solids began in the 1970s and continues to today [14]; the ideal SSE material should have high ionic conductivity (>0.1 mS/cm), low electronic conductivity (<10^−7^ mS/cm), expansive electrochemical windows (>4 V), solid electrochemical stability and high mechanical properties (shear and bulk modulus) [15]. In the past decades, only very few SSE have been able to achieve room temperature lithium ionic conductivity (>10^−2^ S/cm) like that of liquid electrolytes [16]. However, the high ionic conductivity SSE often face various problems such as narrow chemical windows or poor mechanical properties. Under such strict standards, although many materials scholars have done a lot of works in various aspects, it is still a considerable challenge to design SSE that can be commercially applied [17].

With the gradual application of ML in the materials field, scholars have started to use ML in SSE’s screening work. ML has demonstrated its ability to identify high-performance SSE quickly compared to traditional SSE experimental + computational methods. In last decade, many high ionic conductivity SSE have been predicted and some of them have been confirmed by first-principles calculations, which has undoubtedly shortened the experimental prediction period of SSE significantly (referring to Refs. [12,13]). In this review, we will introduce and discuss the recent progress of ML application in SSE from several aspects, such as the acquisition of data sets, selection of suitable descriptors and algorithmic application of training data, respectively, so that more scholars in the materials field, who do not possess knowledge background of ML, can have a more intuitive feeling about ML.

## 2. Data Sets

Data sets are the most fundamental resource for driving ML models and extracting knowledge [9]. Because data sets, also called “big data”, are too much and too complex for traditional humans, the discipline of “material informatics” has been developed to describe how to seek the structure-property relationship [18]. Many material scientists would like to see how to store relevant material information in one “library” that can be retrieved and searched at any time. The first attempts to develop computer coupled phase diagrams and thermochemistry—the CALPHAD database—were born in the 1970s as the enhancement of computational capabilities [19]. After this, with the further development of extensive density flooding theory calculations relying on quantum mechanics and electromagnetism [20,21], researchers also started to narrow the number of experiments for predicting materials using high throughput screening [22,23].

In 2011, the U.S. government officially announced the launch of Materials Genome Initiative (MGI), meaning that a materials discovery paradigm driven by data and information science is gradually shaping. With various efforts to promote materials data worldwide, a large platform of materials data was built up. More and more materials data became openly accessible, gradually forming plentiful materials science database, which became a significant turning point for materials science. Materials databases became the infrastructure of materials discovery platforms [9]. Most current materials databases are implemented based on first-principles calculations, which can accurately calculate various electronic structures and total energy-related data. It can predict the properties at finite temperatures after considering contributions such as the electron-scale vibrations and the hot electron entropy. Within the last few decades, the electronic structure calculation codes have reached a certain level of maturity [24,25]. Current materials databases can be automated to extend first-principles calculations for many compounds with the only limitations of computational resources [26].

**Figure 2:**
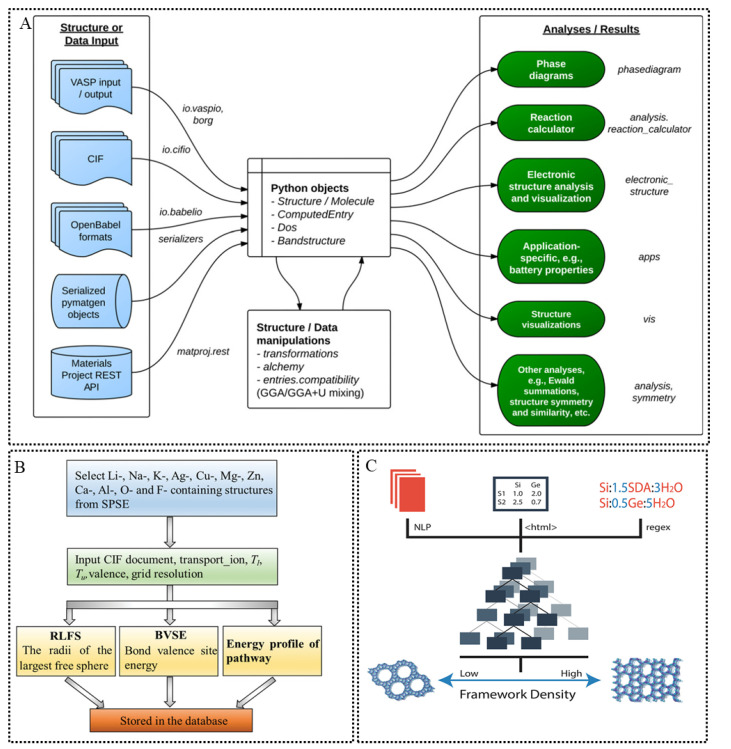
(**A**) Overview of the pymatgen library. Text in italics represents names of Python packages, modules, or classes. Reprinted from Reference [26] with permission from Elsevier. (**B**) The architecture of the ionic transport characteristics database. Reprinted from Reference [29] with permission from John Wiley and Sons. (**C**) Schematic overview of zeolite data engineering, including (1) literature extraction from sources such as NLP from body text, parsing of HTML tables and regex matching between text and tables, (2) regression modeling and (3) zeolite structure prediction. Reprinted from Reference [37] with permission from American Chemical Society.

Appropriate databases can significantly reduce the difficulty of accessing materials data. Table 1 lists the materials databases that have been applied by scholars in materials science for the screening of SSE. Some of these databases provide REpresentational State Transfer (REST), Application Programming Interface (API) [27] interfaces for downloads, such as the Materials Project Database (MP) [28]. In addition, the Python Materials Genomics (pymatgen) library [26]—a powerful open-source python software library (Figure 2A) developed by MP for materials analysis, can obtain valuable materials data and perform complex analysis of materials data through MP’s API interface. Owing to the transport properties of ionic conductors are essential for the performance of SSE, Shi et al. proposed the Matgen database (Figure 2B)—a database containing crystal structure information, ion migration channel connection information and 3D channel maps of over 29,000 inorganic compounds [29]. The Matgen database may be more appropriate in screening ionic properties of SSE.

**Table 1:** Overview of some material databases.

Name	Website	Overview
ICSD	fiz-karlsruhe.de/icsd	Provides information on the crystal structures of all inorganic compounds without C-H bonds, except for metals and alloys [30]
Material project	materialsproject.org	Uses high-throughput computing to uncover the properties of all known inorganic materials [28]
AFLOW	aflowlib.org	The library is mainly composed of chalcogenide data; users can download the whole database [31]
OQMD	oqmd.org	The library is mainly composed of chalcogenide data; users can download the whole database [32]
Computational Materials Repository	cmr.fysik.dtu.dk	Supports the collection, storage, retrieval, analysis and sharing of data produced by many electronic-structure simulators [33]
Crystallography Open Database	crystallography.net	Provides capabilities for all registered users to deposit published and so far unpublished structures as personal communications or pre-publication depositions. Such a setup simultaneously enables the COD database extension by many users [34]
MATGEN	matgen.nscc-gz.cn	Contains crystal structure information, ion migration channel connectivity information and 3D channel maps for over 29,000 inorganic compounds [29]

Ionic conductivity and shear and bulk moduli are complicated and missing in most databases. Therefore, in addition to obtaining datasets from databases and compiling them by themselves based on previous experimental data [35], some scholars in the field of materials science have also attempted to automatically collect material synthesis parameters from tens of thousands of academic publications [36] using text mining, i.e., ML and natural language processing techniques, to integrate and compile them into usable datasets for ML and have successfully performed practical applications [37] (Figure 2C).

## 3. Descriptor

ML models expect the input data to be in the form of letters or numbers. However, the large amount of feature data about the materials we obtain in the original dataset is unsuitable for ML. Therefore, we need to encode and convert material structure data into descriptors (also called feature vectors in ML terminology) that computers can understand through feature engineering. Mapping structure and composition into descriptors that can be easily transported to the ML process is crucial in predicting materials [38]. The input descriptors are more appropriately, the better the ML algorithm can map to the final output data [39]. Depending on the problem under study and the prediction accuracy required, descriptors can be defined as: the higher expected precision needs data-intensive and less conceptual model and laborious learning framework. Therefore, coarser descriptors should usually target a fast and rough initial screening of the material [40].

Descriptors should be able to map the atomic description into the form of a matrix operation [41]. The essential properties must have been the differentiability of atomic shifts and invariance to the fundamental symmetries of physics: rotation, reflection, translation and alignment of atoms of the same species [42]. Descriptors are generally distinguished as global or local descriptors, where global descriptors can usually be used to predict properties related to the whole structure, such as band gaps [43], molecular atomization energies [44], etc. In contrast, local descriptors are generally applied to predict local properties such as adsorption energies [45]. Usually, the screening process for crystalline solids typically considers mainly global properties and secondarily local features [46]. However, for the screening of high ionic conductivity of SSE, many properties have an impact on them, which is difficult to determine when the mechanistic link between descriptors and properties is not clear [47]. To predict the properties of high ionic conductivity and high mechanical strength of SSE, it is generally necessary to construct the corresponding descriptor sets containing several descriptors based on different properties of the dataset and ML algorithms [7,48]. Table 2 lists common descriptors that materials scholars expect to use in the process of screening SSE-related properties using ML and provides a brief description of them.

In the past decades, the primary descriptor in most calculations for SSE remains the structural characteristics of single crystals [52]. However, the size distribution of grains, short-circuiting grain boundaries can lead to inhomogeneous conduction pathways on polycrystalline samples [53], which often depended on experimental conditions such as sintering temperature [54]. The description of volume and grain boundary conductivity is not sufficiently clear. In addition, there are very few reports on how to construct a descriptor about the grain distribution and grain boundaries [55]. For accurate predicting SSE, determining the appropriate descriptors is currently an extreme challenge for experts in this field.

## 4. Construction of ML Model

**Figure 3:**
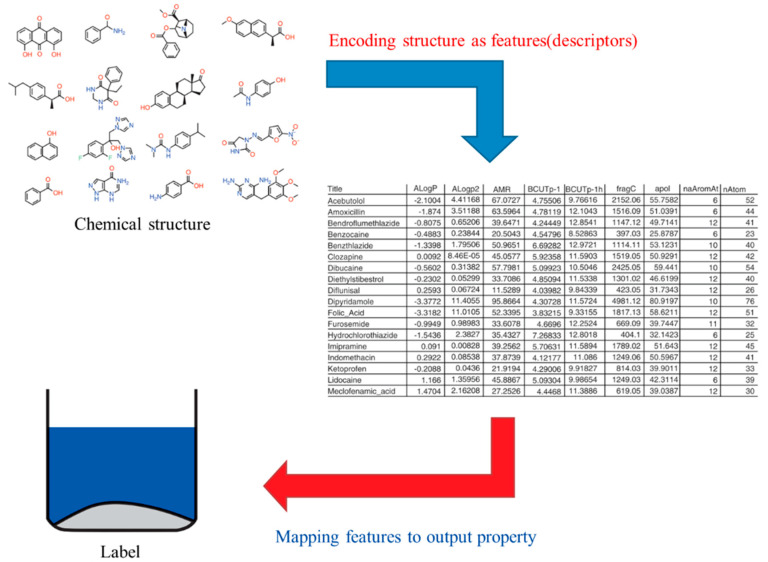
Conceive chemoinformatics as a two-part problem: encoding chemical structure as features and mapping the parts to the output property. The second of these is most often the province of ML. Revised from Reference [56] with permission from John Wiley and Sons.

Appropriate ML algorithms are undoubtedly fundamental in the prediction process. They significantly impact the prediction outcome, but scholars have not found the best method to be applied to all cases so far. The construction of a suitable ML algorithm model is divided into two main stages: the first stage is to encode the data into feature vectors (i.e., descriptors) as model input data and the second stage is to use the algorithm to map the input data [56] on the corresponding desired attributes and we usually refer to the output data of this mapping as labels (Figure 3). By ML, we can find the mapping relationship between features and brands. When there is unknown data input with features but no labels, we can get the titles of the anonymous data by the existing relationship.

In a broad sense, we can divide ML into three categories: supervised learning, unsupervised learning and semi-supervised learning. The main difference between the three is the type and amount of available data.

### 4.1. Supervised Learning Model

Each data in the training set already has features and labels, i.e., it has input data and output data by learning the relationship between input data and output data in the training set. Supervised learning requires a training set and a test set to find patterns in the training set and test them in the test set. Supervised learning can predict output values in continuous quantities (e.g., bulk modulus, bandgap, etc.) or discrete quantities (e.g., crystal structure, etc.) [40] and building models for the former requires regression and the latter involves classification, the exact difference between the two depending on the type of data and the problem posed, respectively. Some computational techniques can be applied to both regression and classification. Supervised learning is like distributing fruits to people, where the fruits are given and the results of the fruit classification (what category each fruit belongs to) are provided as reference answers. A part of the fruits is left as a control test. By doing so, the training is usually adequate. Currently, the main application of the SSE screening process is generally the supervised learning model [57] and the most critical application of ML in the materials domain is also the supervised learning model [58,59].

### 4.2. Unsupervised Learning Model

In contrast to supervised learning models, unsupervised learning uses only feature vectors and not labels, usually unknown in unsupervised learning models. Unsupervised learning models need to reveal the patterns within the data themselves to help find them. Unsupervised learning is the equivalent of assigning a reference standard to a person without giving them a list of similarities to indicate which fruits are in the same category. Unsupervised learning models are generally applied for classification purposes or reduction of the dimensionality of the fingerprint vector. Unsupervised methods solve the problem of being created from sparse datasets. Still, because of this, the accuracy of the data can have a significant impact on the results of unsupervised learning models when applying small dataset construction.

### 4.3. Semi-Supervised Learning Model

The function is generated by combining the data in the training set partly with features and labels and partly with only features in the middle band of supervised and unsupervised learning. The basic rule is that the local characteristics of some labeled data and the overall distribution of unlabeled data are used to obtain acceptable or good classification results [60]. Semi-supervised learning is equivalent to distributing fruits to a person, classifying some of the fruits and letting the person explore the laws to organize the other fruits by himself. Currently speaking, the use of semi-supervised learning in SSE prediction is relatively rare.

## 5. Algorithm Application

According to different data types and quantities, all three ML models are used to construct a predictive SSE model. In most cases, the prediction process for SSE is the same as other materials. Most SSE cases were using the supervised learning model. SSE are mainly divided into different compositions such as oxides, sulfides, halides, etc. The descriptors from various properties of SSE are difference reported in different literatures. Next, we will specifically analyze the ML algorithms that have been applied in the prediction model of SSE.

Kernel methods are a collection of pattern recognition algorithms; the most widely used Kernel methods include support vector machines (SVM) [61] and Kernel ridge regression (KRR). The core of Kernel methods is the use of Kernel functions. The Kernel function is a function that converts the input data into a higher dimensional representation, reducing the computational complexity and making the problem easier to solve. Fujimura et al. [57] used SVM regression to train an ML model with diffusion-related properties. The authors predicted the ionic conductivity of 72 compounds at 373 K, finally predicting that one of them, Li_4_GeO_4_, has the highest ionic conductivity. In this work, the phase transition temperature (T_c_), the diffusivity (D_1600_), the average volume of the disordered structure (V_diss_) and the experimental temperature T were served as independent variables, while the logarithm of the ionic conductivity as the dependent variable. The first-principles calculations were performed iteratively and centrally, which significantly accelerated the prediction process, suggesting potentially superior candidate lithium superionic conductors. The elastic tensor constants of the cubic-phase materials were trained using Kernel ridge regression and gradient lift regression. The interfacial stability between the anode and the SSE can also be used to find potential SSE [62], finally finding high mechanical performance SSE such as LiOH, LiAuI_4_, LiBH_4_, Li_2_WS_4_, etc. These SSE have high ionic conductivity properties while having interfacial stability. Cubuk et al. [63] performed migration learning by SVM using descriptors with physical guidance, which allowed the screening of 20 billion ternary and quaternary Li-containing compounds and proposed some of them as promising SSE candidates.

The main idea of Sparse Gaussian Process Regression (SGPR) is to select a representative subset of the available training data for the Gaussian Process Regression (GPR) approximation model. GPR is a nonparametric model that uses a Gaussian process before regressing the data, in which each point in the continuous input space is associated with a normally distributed random variable. Hajibabaei et al. [64] applied SGPR to hundreds of potential SSE, focusing mainly on ternary SSE and obtained 22 fast Li-ion conductors, four of which have the same set of elements (Li-P-S). In this investigation, it was shown that the models generated using the SGPR method can be more easily combined and can be directly applied to model quaternary composite crystals, an approach that provides a foundation for subsequent studies of SSE with complex elements.

A decision tree is a prevalent classification model representing a mapping relationship between object attributes and values. Each node in the tree represents an object. In contrast, each bifurcation path represents a possible attribute value. Each leaf node corresponds to the entity’s value represented by the way experienced from the root node to that leaf node [65]. Decision trees are often used in integration methods, which combine multiple trees into a single predictive model to improve performance. For example, random forests [66] or rotating forests [67], two algorithms commonly used in the materials field, are attributed to decision tree models. Light Gradient Boosting Machine (LightGBM)-an algorithmic framework that implements gradient descent trees (an iterative decision tree algorithm), has been used to predict mechanically superior electrolytes [68]. With this algorithm, physical properties were found to be the most influential features for predicting mechanical properties (volume, density, space group number and atomic number) and the 17,621 SSE in the database were filtered to obtain 2842 SSE with high mechanical properties. It is believed that this model and other data sets can accelerate finding the best SSE to satisfy the sought mechanical conditions.

The logistic regression model is a generalized linear regression analysis model that focuses on the relationship between the dependent and independent variables. To identify potential superionic structures from a database using training data, Sendek et al. constructed a multivariate predictor of high ionic conductivity from feature vectors. This work utilized a Logistic Regression model (LR) to differentiate and successfully screened 12,831 lithium-containing solid materials to 21 promising structures and proposed a simple atomic descriptor function, which cannot provide predictive power for ionic conductivity alone [35]. Sendek et al. analyzed the misinformation and compiled this information, which is undoubtedly extremely necessary for the prediction of SSE. In addition, new data suggest that halide-based SSE are more likely to meet the requirements of high ionic conductivity and electrochemical stability compared to sulfides and oxides [69].

**Figure 4:**
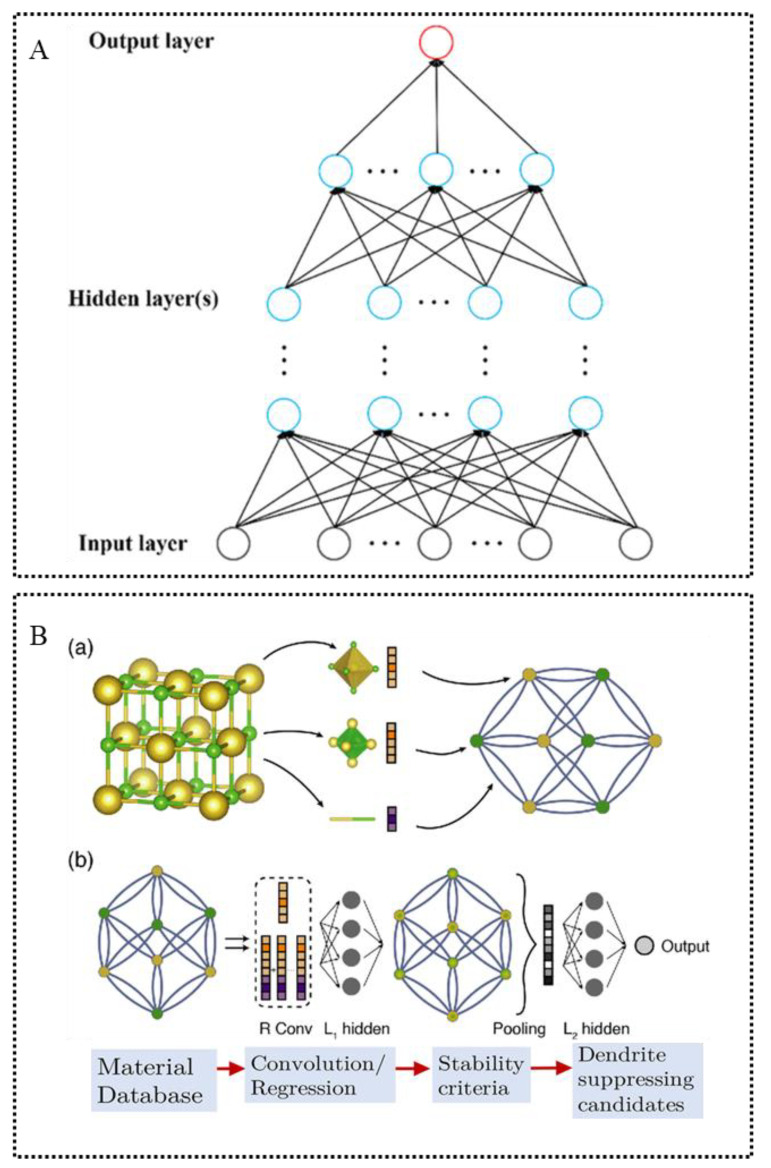
(**A**) Diagram of a typical artificial neural network. The black, blue and red circles indicate input, hidden and output layers. Each circle represents an artificial neuron and arrows indicate connections from the output of one neuron to the input of another. Reprinted from Reference [70] with permission from John Wiley and Sons. (**B**) Illustration of the crystal graph convolutional neural networks and the screening process of high mechanical properties SSE using crystal graph convolutional neural network. (**a**) Construction of the crystal graph. Crystals are converted to graphs with nodes representing atoms in the unit cell and edges representing atom connections. Nodes and edges are characterized by vectors corresponding to the atoms and bonds in the crystal, respectively. (**b**) Structure of the convolutional neural network on top of the crystal graph. R convolutional layers and L1 hidden layers are built on top of each node, resulting in a new graph with each node representing the local environment of each atom. After pooling, a vector representing the entire crystal is connected to L2 hidden layers, followed by the output layer to provide the prediction. Revised from Reference [72] with permission from American Physical Society. Revised from Reference [62] with permission from American Chemical Society.

Neural networks are constructed based on the neural network principle of the human brain in biology, which can mimic the operation of the brain: a large number of neurons (processing units) are interconnected and each connection between two neurons represents a weighted value for the signal of that connection, which is equivalent to the memory of the neural network. The interconnections of neurons from a complete net that processes the input data layer by layer can convert them into a more closely related representation to the output target [70] (Figure 4A). Thus, ANNs have a solid ability to capture complex nonlinear relationships from large-scale datasets, but practical applications are less frequent in screening SSE. Convolutional neural networks [71], which include convolutional computation and have a deep structure, are now frequently used for material prediction. Convolutional neural networks have more layers of neural networks, perform well with more data and can be applied to both supervised and unsupervised learning. For better application in materials, Xie and Grossman proposed a generalized crystal graph convolutional neural network (CGCNN) framework by constructing neural networks on crystal graphs generated from crystal structures [72]. Ahmad et al. successfully screened over 12,000 inorganic solids for shear modulus and bulk modulus using CGCNN, as already mentioned in the previous section, which helped improve the mechanical properties of SSE [62]. The crystal graph convolutional network illustration and the screening process of high mechanical properties SSE using crystal graph convolutional neural network is shown in Figure 4B.

**Figure 5:**
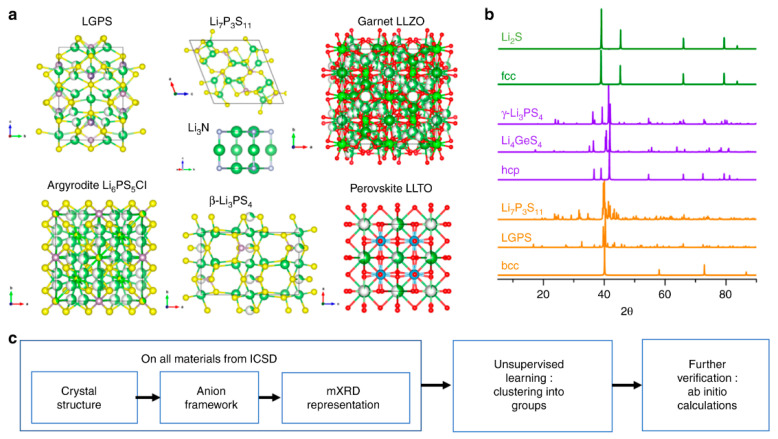
Schematics of the unsupervised discovery of solid-state Li-ion conductors. (**a**) Crystal structures of known Li-ion conductors, showing a large diversity of design and chemistry. (**b**) mXRD patterns of selected materials in comparison to those of ideal fcc (face-centered cubic), hcp (hexagonal close-packed), bcc (body-centered cubic) lattices. (**c**) Workflow of unsupervised learning guided discovery of Li-ion conductors. Reprinted from Reference [73] with permission from Springer Nature.

The clustering algorithm is an unsupervised learning algorithm that requires only data without labeling results. Clustering algorithm brings similar samples together and similarity is defined by distance, with high similarity within groups and low similarity between groups. The models can be clustered into classes. Hierarchical clustering in clustering allowed to successfully distinguish fast lithium conductors from poor lithium conductors [73]. Zhang et al. used a quantitative representation of the complex material structure as input to train an unsupervised model (Figure 5a) and they classified the modified X-ray diffraction (mXRD) using a clustering approach to define each anion lattice and fully capture the anion crystal structure information (Figure 5b). They confirmed that the symmetry and order of the mXRD-encoded anion lattice of SSE are closely related to the ionic conductivity, which led to the prediction of 16 new compounds with high lithium-ion conductivity, a few of which exceed 10^−2^ S/cm. Most of these newly discovered materials are highly different from the currently known fast lithium-ion conductors in terms of chemical composition and structure. It demonstrates the effectiveness of unsupervised learning methods for finding new materials in an extensive range of material spaces and reveals unique structure-property relationships between anion lattices and Li^+^ conductivity in large material spaces. The workflow of unsupervised learning-guided solid-state lithium-ion conductor discovery is shown in Figure 5c.

In addition to the above ML algorithms, several ML algorithms such as k-nearest neighbor (KNN) algorithm [74], Naïve Bayes classifier [75], linear regression (LR) [76] and gradient boosted regression (GBR) [77] have been used in the materials domain. However, there are fewer prediction processes involving SSE, so we will not dwell on them too much. All the algorithms are not independent of each other. The data can be analyzed by comprehensive statistical tests of several algorithms in algorithm modeling [78] to obtain the best results. We can note that in many of the above algorithms, neural networks can learn layer by layer on the input and produce high learning rates, so neural network algorithms are often combined with other algorithms to build prediction models and thus obtain higher accuracy on the data results. The clustering method, which can find complex patterns hidden behind multidimensional data, is well suited for predicting ionic conductivity of SSE, but the clustering method relies more on high-precision data, which is often difficult to obtain and is, therefore, less commonly used than other algorithms.

## 6. Algorithm Optimization

After constructing a model, we may find a significant error when using the trained model for prediction. It is time to optimize our model to reduce the error to the lowest possible level. High bias (underfitting) occurs when the model is not flexible enough to describe the relationship between input and predicted output or when the data is not detailed enough to find patterns. High variance (overfitting) occurs when the model is too complex, the sample size is too small, or other problems such as mislabeling [39]. In simple terms, underfitting is occurred when the data features are not captured better. Thus, the data cannot be fitted well, while overfitting is occurred when the model learns data so thoroughly that the parts of the noisy data are also known. The balancing act between overfitting and underfitting is called the bias-variance tradeoff and is usually controlled by cross-validation (CV) and a more refined dataset design [79]. The basic idea of cross-validation is to group the original data into a training set, a validation set and a test set and then evaluate the accuracy of the model trained in the training set with the data from the validation set, averaging the results of several evaluations as the final evaluation of the model accuracy and using them to adjust the algorithmic model.

## 7. Views and Conclusions

ML has now started to be gradually and thoroughly applied to materials science and has already brought many promising applications to SSE research. In the case of LIB SSE prediction, we can see that ML algorithms perform well: helping researchers extend datasets by text mining from the literatures [36,80], providing new tools for screening SSE with high mechanical properties or high ionic conductivity. In terms of predicting materials, the reduction of computational cycles is undoubted great importance. However, it is undeniable that materials informatics derived from ML is still in its infancy and there are still apparent challenges for materials experts.

The complete process from framing the model to the final prediction is very laborious. ML is a multi-disciplinary discipline and for some materials researchers with little background in computing, there are significant barriers to entering the field. The integration of ML models into modules that can be useful to new scholars to the area is a considerable challenge. Pu et al. [81] proposed an interactive system for experts to select appropriate ML models, representing the progress that some scholars in this area have made.

ML requires a large amount of data for learning to ensure its accuracy. However, many data are limited to hundreds, such as the screening process of SSE. Some data are even qualified to tens, which affects the accuracy of screening results. Little attention has been paid to reports of failure data in this field, but it has to be acknowledged that failure data are also critical [82]. To solve the problem of little learning data from small samples, the learning to learn model has been developed, which called meta-learning [83].

All fields suffer from a reproducibility crisis. The process of not reproducing data from the literature and the need to explore it from scratch due to changes in software versions or default variables can be excruciating for experts in the field. Artrith et al. suggest making complete code or workflows available in public repositories that guarantee long-term archiving [84] so that others and further refined can fully replicate them.

We have reviewed the general process of ML in materials prediction in an easy-to-understand manner and described the latest approaches and specific applications of ML in SSE prediction. Although there are still many challenges in this field, partial solutions have gradually emerged in the literature. Recently, ML has been proposed as a successful model for SSE prediction and can predict desired new materials, suggesting that the use of ML is transformative for materials research. It is still a great challenge to make the models more interpretable for scholars. Undoubtedly, the data-driven materials science will become a significant future research trend. We expect more materials scholars to be aware of this paradigm and pay attention to it.

## Figures and Tables

**Table 2:** Overview of some common descriptors.

Descriptor	Overview
Coulomb matrix (CM)	It represents an atom-by-atom square matrix. The structure is encoded according to the Coulomb force between each pair of atomic charges, in which the off-diagonal element is the Coulomb nuclear repulsion term between atomic pairs [44].
Smooth overlap of atomic positions (SOAP)	SOAP is a translation, rotation and arrangement-invariant descriptor for obtaining the translation, rotation and arrangement of atomic groups, which is the basis for developing various ML interatomic potentials [42].
Diffraction fingerprint	The diffraction fingerprint emphasizes the global characteristics of infinite periodic crystals, which are excited by the properties of the Fourier transform [49].
Topological descriptor	Commonly referred to as path-based fingerprints, chemical structures are encoded according to combinations of atom types and paths between them (e.g., atom-pair fingerprints). They are essentially graph-based descriptors [50].
Quantum descriptors	Based on first-principles calculations. The descriptors calculated from the wave function include energy levels, dipole moments, polarizability, etc. The quantum descriptors are often considered to be more versatile since they better represent the properties, but more difficult and time-consuming to obtain than the other descriptors for the structure [51].
